# Microorganisms for Bioremediation of Soils Contaminated with Heavy Metals

**DOI:** 10.3390/microorganisms11040864

**Published:** 2023-03-28

**Authors:** Victor V. Atuchin, Lyudmila K. Asyakina, Yulia R. Serazetdinova, Anna S. Frolova, Natalia S. Velichkovich, Alexander Yu. Prosekov

**Affiliations:** 1 Laboratory of Optical Materials and Structures, Institute of Semiconductor Physics, Siberian Branch of the Russian Academy of Sciences, Novosibirsk 630090, Russia; 2 Research and Development Department, Kemerovo State University, Kemerovo 650000, Russia; 3 Department of Industrial Machinery Design, Novosibirsk State Technical University, Novosibirsk 630073, Russia; 4 R&D Center “Advanced Electronic Technologies”, Tomsk State University, Tomsk 634034, Russia; 5 Laboratory of Phytoremediation of Technogenically Disturbed Ecosystems, Kemerovo State University, Kemerovo 650056, Russia; 6 Department of Bionanotechnology, Kemerovo State University, Kemerovo 650056, Russia

**Keywords:** agriculture, heavy metals, environmental pollution, bioremediation, *Achromobacter denitrificans*, *Klebsiella oxytoca*, *Rhizobium radiobacter*

## Abstract

Heavy-metal contaminants are one of the most relevant problems of contemporary agriculture. High toxicity and the ability to accumulate in soils and crops pose a serious threat to food security. To solve this problem, it is necessary to accelerate the pace of restoration of disturbed agricultural lands. Bioremediation is an effective treatment for agricultural soil pollution. It relies on the ability of microorganisms to remove pollutants. The purpose of this study is to create a consortium based on microorganisms isolated from technogenic sites for further development in the field of soil restoration in agriculture. In the study, promising strains that can remove heavy metals from experimental media were selected: *Pantoea* sp., *Achromobacter denitrificans*, *Klebsiella oxytoca*, *Rhizobium radiobacter*, and *Pseudomonas fluorescens*. On their basis, consortiums were compiled, which were investigated for the ability to remove heavy metals from nutrient media, as well as to produce phytohormones. The most effective was Consortium D, which included *Achromobacter denitrificans*, *Klebsiella oxytoca*, and *Rhizobium radiobacter* in a ratio of 1:1:2, respectively. The ability of this consortium to produce indole-3-acetic acid and indole-3-butyric acid was 18.03 μg/L and 2.02 μg/L, respectively; the absorption capacity for heavy metals from the experimental media was Cd (56.39 mg/L), Hg (58.03 mg/L), As (61.17 mg/L), Pb (91.13 mg/L), and Ni (98.22 mg/L). Consortium D has also been found to be effective in conditions of mixed heavy-metal contamination. Due to the fact that the further use of the consortium will be focused on the soil of agricultural land cleanup, its ability to intensify the process of phytoremediation has been studied. The combined use of *Trifolium pratense* L. and the developed consortium ensured the removal of about 32% Pb, 15% As, 13% Hg, 31% Ni, and 25% Cd from the soil. Further research will be aimed at developing a biological product to improve the efficiency of remediation of lands withdrawn from agricultural use.

## 1. Introduction

Rapid population growth and industrialization increase human demand for food. Only larger and more productive agricultural areas can provide food security [1,2]. Unfortunately, the ever-growing anthropogenic load affects the productive properties of agricultural soils and their fertility by causing organic matter reduction, nutrient depletion, pollution with heavy metals, pesticides, mineral fertilizers, polycyclic aromatic hydrocarbons, etc. [3,4]. Poor agricultural practices and management of water and land resources cause a drastic decline in agricultural soil quality and induce disastrous economic losses [5,6,7]. This situation poses an urgent task of reducing the degradation rate of disturbed farming lands and increasing its restoration rate. This task is one of the 17 main goals of sustainable development defined by the United Nations through 2030 [8].

Global agricultural communities are particularly concerned with the current heavy-metal contamination of agricultural lands. Heavy metals are highly toxic compounds that can persist in soils for a long time. Cd, Pb, Zn, and Cu enter agricultural soil with fertilizers, including organic ones. However, arsenic and mercury can also mix with agricultural soil if the field is located near industrial enterprises [9,10,11]. Moreover, heavy metals are prone to bioaccumulation. They are known to accumulate in agricultural crops, thus changing their biochemical and physiological processes [12]. Not only do they reduce plant productivity, but they also lead to necrosis of plant tissues, if allowed to reach high concentrations [13,14]. Heavy metals enter living organisms with agricultural products. As a result of biomagnification, they accumulate until they become a serious threat to the health of animals and people [15,16].

Anthropogenic, or technogenic, soil contamination with metals occurs as a result of the activities of mining enterprises, the burning of enterprise waste, oil and oil product spills, transport emissions, industrial and domestic waste dumps, etc. [17,18]. Left unattended, industrial waste causes heavy metals to migrate to agricultural areas as a result of heavy rains, natural erosion, or microbial activity [19,20].

Agriculture is another source of heavy-metal contamination of soil. For example, excessive Cd, Cu, and As can be associated with a severe overuse of chemical fertilizers. Cd is an important metal component of phosphate ore, which is used in phosphate fertilizers, and may also contain As [21]. Herbicides and pesticides also contribute to the pollution of agricultural soils. For instance, Defarge et al. proved that pesticides contain Cr, Co, Pb, and Ni [22]. Bai et al. reported that the content of Co, Zn, and Cu in greenhouse soils correlates with the cultivation time. These pollutants probably accumulated due to fertilizers [23].

Heavy metals can be removed from soil by physicochemical or biological methods. Physical and chemical methods include replacement of the soil layer, electrokinetic removal, thermal treatment, soil washing, vitrification, and chemical treatment with lime, phosphate compounds, or organic compounds [15]. Each group of methods has its own advantages and disadvantages. Physicochemical methods are efficient and fast while being expensive and labor-consuming. Eventually, they can cause drastic changes in soil quality indicators. Therefore, physicochemical methods provide no optimal solution in agriculture [15]. Biological treatment is a good alternative because it is economical and environmentally friendly. Biological methods rely on plants (phytoremediation) and microorganisms (bioremediation) [24].

Bioremediation has attracted a lot of scientific attention in recent years. Its mechanisms are based on redox transformations, absorption, and changes in the reaction of the medium. Currently, the most common methods of microbial removal of heavy metals are biosorption–bioaccumulation, production of biosurfactants, bioleaching, oxidation–reduction, biovolatilization, biomineralization, etc. [25]. Microorganisms are able to develop protective systems to avoid negative effects; however, most heavy metals destroy the membranes of microbial cells. Therefore, the ability of microorganisms to remain viable under the influence of heavy metals in the restoration of disturbed areas is of decisive importance [26]. According to literature data, it is possible to use Bacteroidetes and Firmicutes for As-contaminated areas. Their abundance positively correlates with this pollutant in contaminated areas. It is also noted that proteobacteria are resistant to high concentrations of Zn, as well as Pb [27].

The toxicity and mobility of most heavy metals, e.g., Cu, Se, Pb, Cr As, or Ni, depend on the oxidation degree [28]. Using these pollutants as food sources, microorganisms change their redox potential [29]. Yang et al. showed that stimulation of native microorganisms reduced Cr (VI) to Cr (III). Its carcinogenic and mutagenic properties make hexavalent Cr more toxic than trivalent. When ingested with food, it causes respiratory problems and allergies [30]. Under the stress caused by heavy metals, some microorganisms are able to secrete extracellular polymeric substances, such as polysaccharides, proteins, and lipids containing numerous binding sites that can bind heavy-metal ions. As a result, heavy metals are adsorbed on the surface of the cell wall. For example, *Klebsiella pneumoniae* Kpn555 isolated from coffee waste was found capable of Pb bioaccumulation, and its maximal biosorption capacity was 475 mg/g. In some cases, metals accumulate intracellularly in the cytoplasm, where they are transformed under the action of enzymes [31]. The biosorption abilities of microorganisms can accelerate *ex-situ* remediation. Soil microbial communities are stable and maintain various ecological processes, e.g.,:

- Nutrient cycle [32];
- Organic carbon balance and degradation [33];
- Prevention and treatment of plant diseases [34].

Some native microbial strains can reduce the concentrations of heavy metals in soil. For example, *Aspergillus niger* M1DGR, which was isolated from the industrial soil of Hattar, Pakistan, demonstrated extremely high bioaccumulation; it was able to extract 98% Cd and 43% Cr [35]. *Stenotrophomonas rhizophila* JC, isolated from the contaminated soil of Jinchang (China), was able to remove 76.9% Pb and 83.4% Cu [36].

It should be noted that the most promising is the isolation of microorganisms from technogenically disturbed soils that are subjected to heavy-metal contamination for a long time. Such microorganisms are resistant to aggressive environmental conditions and in the process of adaptation are able to acquire the necessary destructive properties.

The joint use of microorganisms and plants is also promising. It is well known that soil microorganisms can affect the mobility and bioavailability of heavy metals by dissolving metal phosphates, releasing chelating agents, and causing redox changes [37]. In addition, some bacteria are able to synthesize phytohormones, having a positive effect on plant growth and development. For example, *Rhizobium pusense* KG2 is reported to be able to immobilize Cd^2+^ in soil, stimulate plant growth, and improve plant resistance to Cd [38].

The most promising for the restoration of agricultural soils was the use of meadow clover (*Trifolium pratense* L.). It is widely used as a high-quality fodder, green manure plant, and soil and water conservation plant. Literature evidence suggests that *T. pratense* has high photosynthetic and antioxidant activity. In addition, most of the heavy metals are concentrated in the root system, indicating that it may have a good potential to resist heavy-metal toxicity and promote effective detoxification of contaminated sites [39].

The purpose of this study is to create a consortium based on microorganisms isolated from technogenic sites for further development in the field of soil restoration in agriculture.

## 2. Materials and Methods

**Figure 1:**
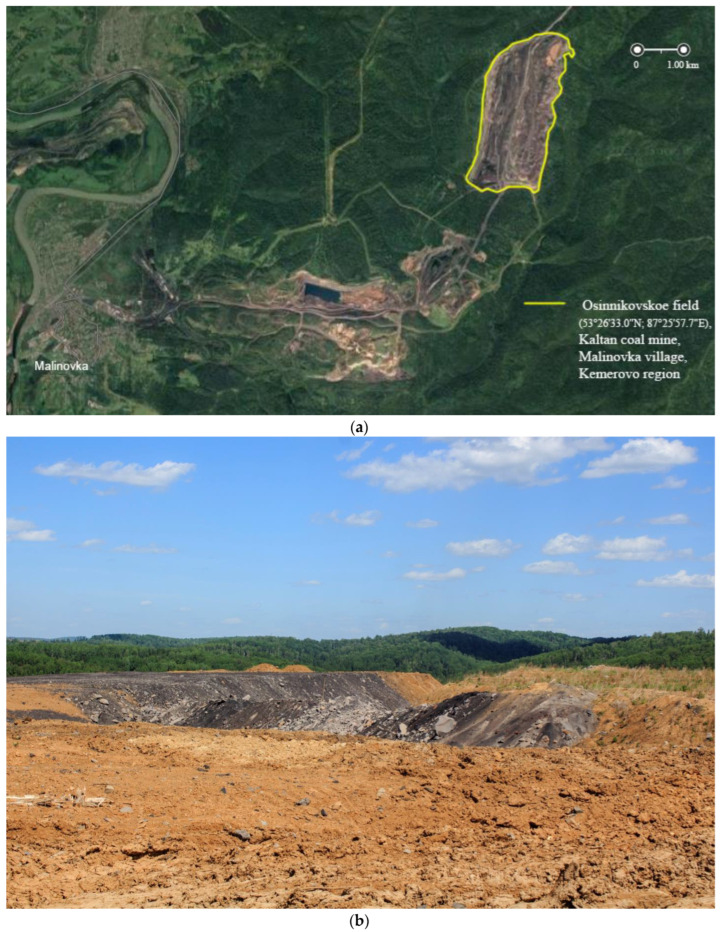
Photo of the Osinnikovskoye coal deposit: (**a**) public cadastral map, (**b**) snapshot of the field.

For the study, samples of technogenically disturbed soils were used, taken on the territory of the Osinnikovskoye deposit (53°26′ N; 87°25′ E) of the coal company OJSC Kuzbassrazrezugol, a branch of the Kaltan coal mine (Figure 1). The dump was laid in the village of Malinovka, Novokuznetsk district, Kemerovo region (Russia). The area of the recultivated territory is 1.2 km^2^. The site is located in a mountainous taiga area. The granulometric composition of soils refers to heavy loams. On the surface of the sites, a significant proportion is occupied by the weathering products of rocks.

The sampling of technogenic soils was carried out in accordance with GOST 17.4.4.02–2017 [40]. The microorganisms were isolated on a liquid nutrient medium that contained heavy metals. The medium included 0.4 g KH₂PO₄, 3.0 g (NH₄)₂SO₄, 3.0 g Na₃C₆H₅O₇, 0.01 g CuSO₄, 0.005 g ZnSO₄, 0.001 g FeSO₄, 0.2 g CdCl_2_, 0.5 g MgSO₄, 0.1 g MnSO₄, 8.0 g peptone, 5.0 g maltose, 20.0 g agar-agar, and ≤1 L water. We added 1 g of soil to 100 mL of sterile medium and cultivated it in an LSI-3016A shaker-incubator (Daihan Labtech, Namyangju, Republic of Korea) at 100 rpm and 25 °C for 24 h. To obtain pure isolates, 1 mL of the liquid culture was sown by the surface method on a solid medium with the abovementioned composition. It remained in a TSO–1/80 SPU thermostat (Smolensk SKTB SPU, Smolensk, Russia) at 25 °C for 24 h. To determine the morphological features, single colonies were obtained by seeding the isolates by the streak plate method on plain agar. The biochemical profile of the cell wall was described by the Gram method [41]. The microscopy of the samples involved an Axio Scope A1 microscope (Zeiss, Jena, Germany).

To select the most promising strains, the samples were examined for the ability to absorb heavy metals from the nutrient medium while maintaining the rate of biomass growth in comparison with the control sample (there were no heavy metals in the medium). Promising microorganisms were identified and tested for biocompatibility. The results of the study were used to form consortiums. Next, the designed consortia were studied for their ability to absorb heavy metals and their mixtures. After the development of a consortium for soil purification, the ability of the consortium to influence the growth and development of plants (due to the synthesis of the phytohormone–indoleacetic acid) was studied. Further, for the most promising consortium, the cultivation optimum was determined, and its ability to increase the efficiency of heavy-metal extraction from the soil medium by the phytoremediation method was determined. A detailed description of the methods used is provided below.

The uptake potential of Pb, Ni, As, Cd, and Hg was tested on inoculums of isolated microorganisms in beef extract broth. The working solutions metal salts Na_3_AsO_4_, Pb(NO_3_)_2_, Ni(NO_3_), Cd(NO_3_)_2_, and Hg(NO_3_)_2_ had a concentration of 1 mg/mL, 10 mL of which was added to 90 mL of the beef extract broth. The control sample contained no metal. The flasks were kept in an LSI-3016A shaker-incubator at 100 rpm and 25 °C for 5 days. The residual heavy metal in the medium was measured on an atomic emission spectrometer with microwave plasma ICPE-9820 (Shimadzu, Kyoto, Japan). Standard metal ion reductions are used as a reference sample: cadmium No 7874-2000 MSO 0299:2002, lead No 7877-2000 MSO 0302:2002, arsenic (III) No 7996-2001 MSO 0581:2003, nickel No. 7873-2000 MCO 0298:2002, purchased from Ekros-Analitika, Russia. Relative error, at *p* = 0.95 is 1%. The culture liquid was centrifuged at 8000 rpm for 15 min, and 1 mL of concentrated HNO₃ was added to 9 mL of the supernatant. The resulting mix was subjected to hydrolysis; first, it was kept at 180 °C for 35 min, and then it stayed at room temperature in a microwave reactor for 10 min [42]. After that, 0.2 mg/L working solutions of Pb, Ni, As, Cd, and Hg were added to 100 mL of beef extract broth to determine the absorption potential of the mix. After sterilization, we introduced the microbial suspension into the medium. The cultivation and content analysis involved the conditions described above.

To study the biomass accumulation, the culture liquid was centrifuged at 8000 rpm for 15 min. The resulting biomass was kept in a VD 23 BINDER oven (Binder, Neckarsulm, Germany) at 60 °C until constant weight. The biomass accumulation was determined by Equation (1) [43]:

(1) $$\mathrm{Biomass}\;\mathrm{accumulation}\;=\;\frac{m\;(\mathrm{dry}\;\mathrm{biomass}),\;g}{V\;(\mathrm{medium}\;\mathrm{volume}),\;L}$$

The isolates were identified using a Vitek 2 Compact automatic microbiological analyzer. The strains were cultivated on Columbia agar with blood at 25 °C for 18–72 h. Then, we prepared a microbial suspension of 2.70–3.30 by the McFarland density scale. The optical density was determined using a DensiCHEK plus densimeter (Bio-Merieux, Marcy-l’Étoile, France). The suspension was added to GN cards, which can identify a wide range of Gram-negative microorganisms [44].

The biocompatibility test relied on the co-cultivation method. The microbial cultures were grown on beef extract broth at 25 °C for 18–24 h. The samples were centrifuged at 5000 rpm for 5 min. The test culture was evenly applied to a Petri dish with plain agar, and the supernatant was added to 4 mm wells. The thermostating took 24 h at 25 °C. If the test culture closely approached the well and no zone of inhibition was observed, then the microorganisms were marked as biocompatible [45].

The amount of indole-3-acetic acid synthesized by the consortiums was determined according to the method developed by Sarmiento-Lopez et al. [46]. According to this method, 0.1% L-tryptophan and 5% of the bacterial consortium were added to the beef extract broth. The mix was thermostatted for 48 h. After that, the biomass was separated by centrifugation at 10,000 rpm for 5 min. To prepare the Salkowski reagent, we dissolved 0.1 g of FeCl_3_ in 100 mL of 50% H_2_SO_4_. Subsequently, we mixed 1 mL of the resulting solution with 1 mL of the supernatant and kept it at room temperature for 30 min until the solution turned pink. The optical density of the resulting solution was measured on a spectrophotometer at 535 nm. The amount of indole-3-acetic acid was determined from the calibration curve of the standard solution. Indole-3-butyric acid was registered by HPLC using a Shimadzu LC-20AD chromatograph (Shimadzu, Kyoto, Japan) according to the Martinez-Morales method [47].

The sampling procedure involved ethyl acetate. The sample was dried and dissolved in 2 mL of methanol. The next stage involved the optimal parameters for consortium cultivation. To make the environment acidic, we added 1 N HCl to the beef extract broth. To obtain an alkaline environment, we added 1 N NaOH to the beef extract broth. The reaction range of the medium varied from 3.0 to 9.0 in increments of 1 [48]. The optimal temperature was determined during cultivation in the liquid nutrient medium. The temperature range was 20–45 °C in increments of 5 °C.

To study the ability of the consortium to increase the efficiency of phytoremediation, an approbation was carried out on clover. For this, a universal primer was used. The soil was distributed in Petri dishes, after which it was added to each solution of salts of heavy metals: Pb, Ni, As, Cd, and Hg [49,50] at a concentration of 250 mg/kg in a volume of 20 mL. After that, the soil was mixed and left overnight for a better distribution of metals in the soil.

In order to determine the most effective seed treatment option by the consortium, red clover (*Trifolium pratense* L.) seeds were prepared in two ways:

1. Seeds were soaked in a consortium of different concentrations, followed by watering;
2. Seeds were soaked in water, then watered with a consortium with different concentrations.

In the first case, clover seeds were soaked in 5 mL of a consortium of microorganisms at concentrations of 1.5 × 10^−2^ and 2.5 × 10^−2^ McFarland Standards for 24 h at a temperature of 5–8 °C. In the second, clover seeds were soaked in 5 mL of water for 24 h at a temperature of 5–8 °C [51].

After treatment, the seeds were sown in Petri dishes, 10 pieces at a depth of 1 cm [52]. Seeds were irrigated every other day. Seeds treated by the first method were watered with water in the amount of 20 mL per Petri dish. Seeds treated with the second method were watered with solutions of consortia of microorganisms with a concentration of 1.5 × 10^−2^ and 2.5 × 10^−2^ McFarland Standards in the amount of 20 mL per cup.

The control samples were seeds soaked in water for 24 h at a temperature of 5–8 °C, which were watered with water in a volume of 20 mL per Petri dish every other day.

Germination was carried out for 10 days at a temperature of 18–25 °C and a relative humidity of 80% [53]. After the specified period, the plants were removed from the soil. The soil cleared of plant residues was examined for the content of heavy metals.

Determination of the content of the residue of detected metals in the soil (after 10 days of growing clover) was carried out on an atomic emission spectrometer using ICPE-9820 plasma (Shimadzu, Kyoto, Japan) according to a similar method presented in the work of Austin Harris and co-authors [54].

Each experiment was performed in triplicate, and the results of statistical data analysis were expressed as the arithmetic mean ± standard deviation. To analyze the results of the experiment using the consortium in phytoremediation, Student’s *t*-test was used. Data processing was carried out using the package Microsoft Office 2007 (12.0.6612.1000) SP3 MSO (12.0.6607.1000) (Microsoft corporation, Redmond, DC, USA).

## 3. Results

In samples of technogenically disturbed soils, 10 pure microbial cultures were isolated, which turned out to be resistant to heavy metals. The cultural and morphological features are listed in Table 1.

**Figure 2:**
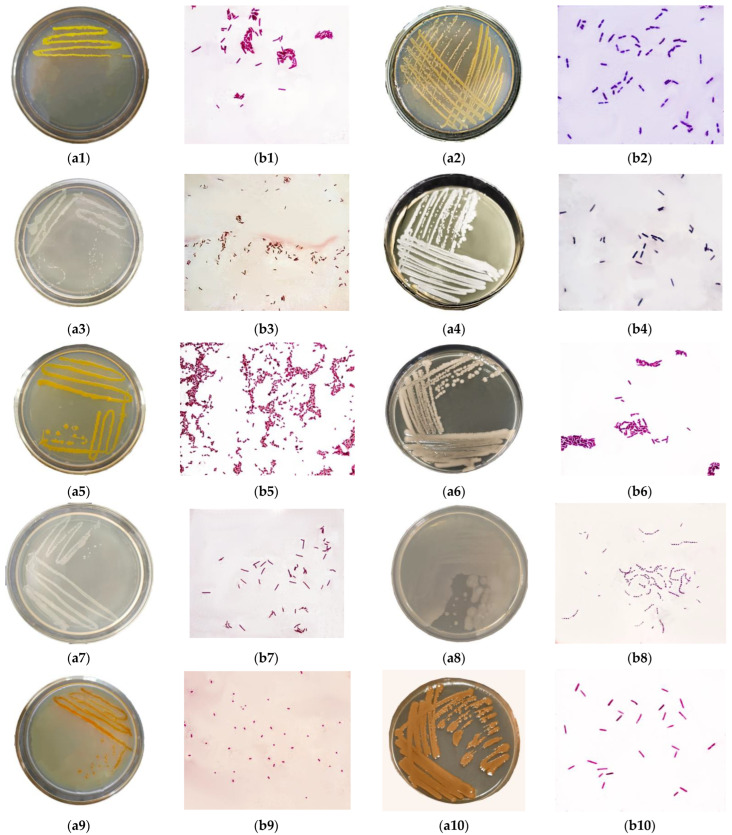
Cultural and morphological properties of isolates: (**a1**–**a10**)—the growth of the studied microorganisms in a Petri dish on the MPA medium, (**b1**–**b10**)—appearance of isolate under a microscope, ×100.

The growth of the studied microorganisms on Petri dishes is shown in Figure 2.

The isolated microorganisms were represented mainly by Gram-negative bacilli. Their location relative to each other was either single or paired, with the only exception of sample 8, where the cocci were located in chains as streptococci. Some colonies were stained orange, yellow, light brown, or light beige, e.g., Samples 1, 2, and 5 were yellow. Samples 4 and 6 had the largest colony diameter of 2.1–2.8 mm and 2.0–3.5 mm, respectively. Table 2 describes the ability of microorganisms to remove certain heavy metals and accumulate biomass. Pb removal varied from 22.04 mg/L to 74.26 mg/L; Samples 1, 3, and 7 demonstrated the highest activity. Most microorganisms utilized ≥25 mg/L As from the nutrient medium, except Isolates 8 (24.57 mg/L) and 9 (10.36 mg/L). As for Hg, the best results belonged to Isolates 3 (73.08 mg/L) and 9 (64.38 mg/L).

The isolated microorganisms also showed excellent Ni extraction results of at least 36.08 mg/L. Isolates 2, 5, and 9 were the least effective samples in removing Cd with 11.84–19.14 mg/L. As for biomass accumulation, heavy metals usually inhibited the cell accumulation in the medium. Nevertheless, most samples with good metal removal rates demonstrated a very insignificant difference with the control sample, which contained no heavy metals (Table 3). We selected those isolates that were able to remove at least 40 mg/L of each individual metal and with a biomass accumulation rate that differed from the control by ≤25%. These were microorganisms in Samples 1, 3, 6, 7, and 10. We used a Vitek 2 Compact automatic microbiological analyzer to define the microorganisms in Isolates 1, 3, 6, 7, and 10 by their physical and biochemical profile. Isolate 1 proved to be *Pantoea* sp. with a 98% probability; Isolate 3 was *Achromobacter denitrificans* (99%), Isolate 6 was *Klebsiella oxytoca* (99%), Isolate 7 was *Rhizobium radiobacter* (98%), and Isolate 10 was *Pseudomonas fluorescens* (99%). Table 4 demonstrates their biochemical characteristics.

The isolates showed good prospects for further research and could be part of a consortium for the purification of heavy-metal-contaminated soils. To enter the consortium, the microorganisms were tested for biocompatibility (Table 5).

**Figure 3:**
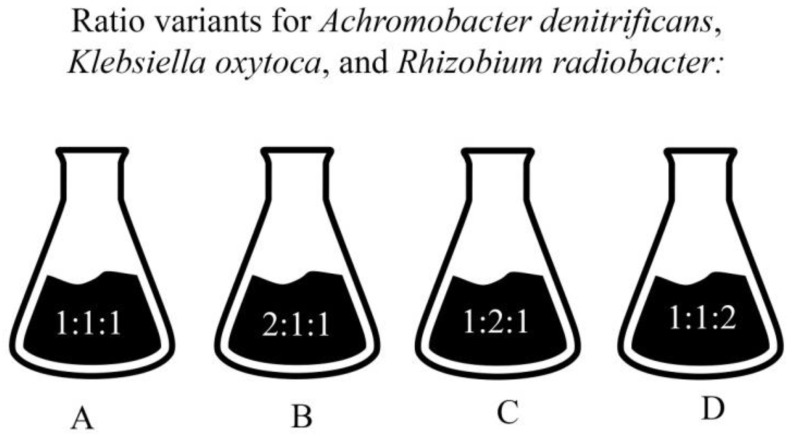
Consortium variants, where (**A**–**D**) is the name of the consortium with the corresponding concentration of microorganisms *Pantoea* sp. *Achromobacter denitrificans*, *Klebsiella oxytoca*, and *Rhizobium radiobacter*.

*Pantoea* sp. and *Pseudomonas fluorescens* could not be used together in one consortium. *Pantoea* sp. inhibited *Achromobacter denitrificans* and *Rhizobium radiobacter*, but exhibited active co-growth with *Klebsiella oxytoca* and *Pseudomonas fluorescens*. *Pseudomonas fluorescens* also did not enter the prospective consortium because it showed no signs of growth except when accompanied by *Pantoea* sp. *Achromobacter denitrificans*, *Klebsiella oxytoca*, and *Rhizobium radiobacter* did not inhibit each other and promoted each other’s biomass accumulation. These microorganisms also showed active growth together in one medium. Figure 3 illustrates various consortium options based on the biocompatibility tests.

The selected microorganisms were turned into four different consortiums. The ratio of *Achromobacter denitrificans, Klebsiella oxytoca*, and *Rhizobium radiobacter* was 1:1:1 in Consortium A, 2:1:1 in Consortium B, 1:2:1 in Consortium C, and 1:1:2 in Consortium D, respectively. The resulting consortiums were tested for their ability to remove individual heavy metals and accumulate biomass (Table 6 and Table 7).

Consortium D had the highest (91.13 mg/L) Pb removal results. It also showed good results for Ni 98.22 mg/L. However, it demonstrated a significant decrease in biomass accumulation on a nutrient medium with mercury. Its heavy-metal removal data ranged from 56.39 mg/L to 98.22 mg/L. Hg proved to be the least extractable heavy metal. The Hg-contaminated environment also had a significantly low biomass accumulation. Consortium B was able to remove the largest amount of Hg (63.90 mg/L) from the nutrient medium. For other metals, its results varied from 38.46 to 82.84 mg/L. As removal was also poor in most of the consortiums. Only Consortium D was able to utilize ≥50% As. However, the biomass increase was quite sufficient in all the samples. Therefore, the low As removal was not associated with low cell viability. The Cd extraction ranged from 47.13 to 94.50 mg/L, with Consortium C being the most efficient one. We studied the ability of the consortiums to remove composite pollutants by measuring the destruction level of heavy metals in the nutrient media (Table 8).

Consortium D proved to be the most promising variant in the treatment of composite pollution with its 47.33–83.26 mg/L. In general, the purification of composite heavy-metal contamination was less effective than in cases with a single pollutant. Cd removal, however, creased by 26.87 mg/L in a composite environment. Consortium B with its 50% showed the worst results for purification of composite heavy-metal pollution. All the consortiums showed no significant decrease in biomass accumulation during cultivation in media with composite contamination. Currently, a large number of studies feature the joint use of phyto- and bioremediation methods. Possibly, some microorganisms could both remove heavy metals from the environment and increase the survival rate of plants. Consortium D and its phytoremediation prospects were studied based on its ability to synthesize phytohormones. Table 9 describes the ability of consortiums to synthesize indole-3-acetic and indole-3-butyric acids.

All the consortiums were capable of synthesizing phytohormones. The synthesis of indole-3-acetic acid varied from 10.12 (Consortium C) to 18.03 (Consortium D) μg/mL of C nutrient medium. The synthesis of indole-3-butyric acid ranged from 0.63 to 2.02 μg/mL of nutrient medium. Consortiums A and D were the leaders in the production of this phytohormone. Thus, the consortiums were effective additional agents for the phytoremediation method. The constant impact of heavy metals on a plant can lead to a decrease in the level of IAA in plant roots and affect the overall growth of plants. Due to this, rhizosphere microorganisms that produce phytohormones are able to prevent the negative consequences of environmental stress [55].

**Figure 4:**
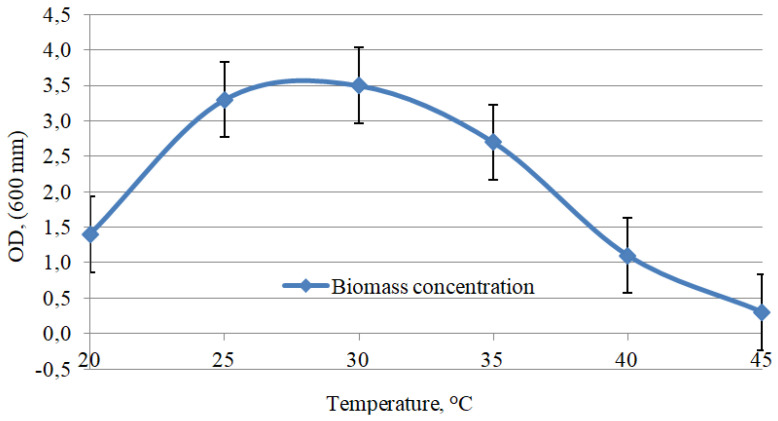
Effect of temperature on biomass accumulation: Consortium D.

**Figure 5:**
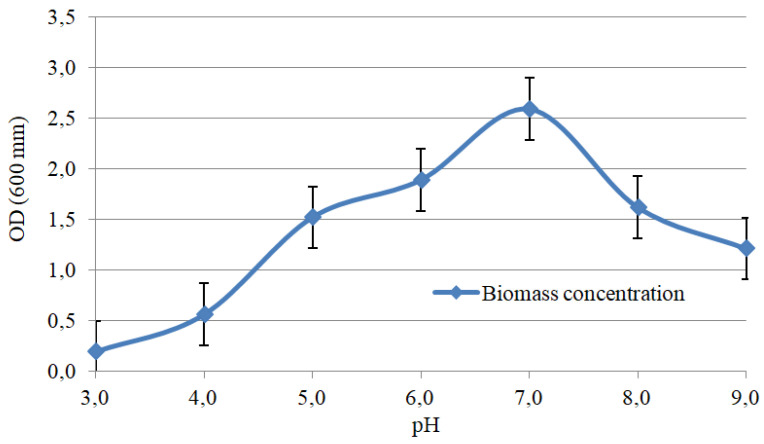
Effect of acidity on biomass accumulation: Consortium D.

Extra biomass contributes by boosting the removal of heavy metals from the environment because cells, e.g., biosurfactants, synthesize active substances, and the binding area between cells and heavy metals increases. In addition, pH and temperature are important growth factors for bacteria. If selected properly, these parameters improve bioremediation and reduce the cost of industrial production. Figure 4 and Figure 5 illustrate the optimal cultivation parameters. The selection of optimal cultivation parameters was carried out for the most promising Consortium D. The consortium is the leader in the removal of individual Pb, Ni, As, and Cu contaminants. The consortium is also the most effective in relation to mixed heavy-metal pollution, and is the leader in the synthesis of indole-3-acetic and indole-3-butyric acids. The selection of optimal cultivation parameters for Consortium D is shown in Figure 4 and Figure 5.

Temperatures ≥35 °C had a negative impact on biomass production. A temperature of 20 °C also inhibited the microbial growth. The optimal cultivation temperature was 25–30 °C.

Consortium D demonstrated low biomass accumulation in both strongly acidic and strongly alkaline environments. While pH = 3 caused no biomass accumulation, at pH = 9, bacterial growth was 1.21 OD (600 mm). pH = 7 was the optimal reaction of the medium on the growth of Consortium D (Figure 5). The data obtained determined the application scope for Consortium D; it cannot be applied in strongly acidic soils because high acidity inhibits cell viability, but alkaline soils could also reduce its effectiveness. The alkaline reaction of the medium was reported to have a negative effect on the electrostatic attraction between heavy metals and the bacterial surface [56]. Since Consortium D not only has the ability to remove heavy metals, but also synthesizes phytohormones that promote plant growth and development, its use in combination with phytoremediation methods is most promising.

In addition, literature data indicate that some microorganisms that are resistant to heavy metals are able to enhance or suppress the ability of plants to absorb metals [57,58]. To evaluate the effectiveness of the constructed consortium in increasing the efficiency of soil phytoremediation, testing was carried out in laboratory conditions on shifts of clover (*Trifolium pratense* L.). The results of the study are presented in Table 10.

According to the data obtained, the most effective way to use Consortium D is pre-sowing seed treatment (soaking). In this case, it is most rational to use a concentration of 2.5 × 10^−2^ according to the McFarland standard. With this processing, the results correspond to the statistical difference for Student’s *t*-test (*p* < 0.05). This method ensures the removal of about 32% Pb, 15% As, 13% Hg, 31% Ni, and 25% Cd from the soil. It should be noted that the rate of removal of heavy metals from the soil is much lower than from the nutrient medium, which is consistent with the data obtained by other researchers [59]. Despite this, when seeds were treated with Consortium D, an increase in the efficiency of phytoremediation was observed compared to the control results (soaking in water and watering). So, when seeds were soaked at a consortium concentration of 2.5 × 10^−2^, the removal of metals increased by Pb 16%, As 10%, Hg 11%, Ni 17%, and Cd 17%. It can be assumed that the use of Consortium D contributes to the better survival of plants under conditions of stress caused by heavy metals. So, in the control samples, the average survival rate of seedlings was about 50% of those planted. The use of the consortium made it possible to achieve plant survival in the range of 70–90%. However, to confirm this hypothesis and obtain statistically significant results, it is necessary to conduct studies with a large number of repetitions. Thus, the use of the consortium to clean up soils with mixed contamination with heavy metals will increase the rate of recovery of disturbed areas. In addition, the consortium can have a positive effect on the survival of plants, but this requires additional research.

## 4. Discussion

The data obtained were consistent with the results obtained by other scientists. For example, *Achromobacter* has been used to bioremediate soils contaminated with heavy metals. *Achromobacter* sp. L3 can immobilize and remove divalent cadmium [60]. Ni et al. showed that K. *Oxytoca* isolates are resistant to such heavy metals as Cu^2+^ (84.8%), Pb^2+^ (80.8%), Cr^3+^ (66.4%), Zn^2+^ (66.4%), and Hg^2+^ (49.6%) [61]. According to *Alboghobeish* et al., some strains of *K. oxytoca* isolated from industrial wastewater were resistant to Ni^2+^ [62]. Some representatives of *Rhizobium* were resistant to high concentrations of heavy metals. They also had good nitrogen-fixing capacity and thus improved plant growth, allowing them to be used as biofertilizers [63,64].

The ability of microorganisms to intensify plant growth and produce growth-stimulating substances is widely covered in the scientific literature. Thus, in a study by Mogal et al., leguminous plants treated with *Rhizobium* spp. observed an increase in the content of indoleacetic, indolebutyric, gibberellic, and other acids in the roots [65].

The ability to produce phytohormones in *Achromobacter denitrificans* is also confirmed. In a study by Singh et al., this microorganism showed the ability to synthesize indoleacetic acid. In addition, the ability of this microorganism to produce siderophores, ammonia, and organic acids was noted [66]. Phytostimulating activity was also noted in bacteria of the genus *Klebsiella*. For example, Mitra et al. reported that *Klebsiella michiganensis* produced indole-3-acetic acid and also carried out phosphate solubilization and nitrogen fixation [67].

Thus, a consortium including *Achromobacter*, *Rhizobium*, and *Klebsiella* may have prospects for application in agriculture, not only contributing to the removal of heavy metals from contaminated soils, but also stimulating the growth of plants in the disturbed area.

Consortium D (presented in this paper) is designed to be effective in heavy-metal removal due to its ability to operate under mixed contamination conditions. Currently, most of the developed microbial preparations are aimed at removing one or two, and less often three, metals [68,69,70]. Consortium D is active against five heavy metals at once, which include Pb, As, Hg, Ni, and Cd.

The elimination of mixed pollution by heavy metals is an urgent problem that is widely reported in the scientific community. For example, Liu et al. have established the effectiveness of manganese-oxidizing bacteria against mixed pollution of As, Pb, and Cd. According to the presented data, the removal of As, Pb, and Cd in the composite phase of polluted water varied from 22 to 35% [59]. This is less efficient than the use of the Consortium D developed in this study. The absorption of metals from the aquatic environment for it ranged from 47 to 83%. In addition, the microorganisms included in the consortium are native, isolated from the technogenically disturbed territories of the region. Accordingly, they are adapted to the soil and climatic conditions of the restored areas.

Due to the ability of microorganisms to remove heavy metals from the environment, as well as to intensify the growth and development of plants, it is promising to use a combination of phyto- and bioremediation methods. This is confirmed by the data of modern scientific literature. It is reported that the inoculation of Miscanthus sinensis A. with the Pseudomonas koreensis AGB-1 strain increased the solubilization of heavy metals, as well as their availability in the plant rhizosphere. In addition, a decrease in oxidative stress from heavy metals was observed, as well as an increased increase in biomass in Miscanthus sinensis A [71]. Durand et al. found that plant inoculation with PGPR Variovorax NB24 resulted in increased nickel accumulation in roots and aerial parts [72].

Further research in the field of bioremediation of heavy metals will explore the mechanisms of removal of pollutants from the environment. A better understanding of the processes will improve cleaning efficiency. The consortium created within the framework of this work has great prospects not only in soil cleanup. His further research can be directed to the development of sorption systems by the method of immobilization on a solid support, which is currently a promising direction [56]. Adsorption systems obtained by this method can be used to treat industrial wastewater, as well as industrial water contaminated with heavy metals. However, further research is required in this area in order to select a carrier that allows reaching the maximum degree of purification and is easily removed from the treated areas.

## 5. Conclusions

This study featured ten pure microbial strains isolated from technogenically disturbed soils. *Achromobacter denitrificans*, *Klebsiella oxytoca*, and *Rhizobium radiobacter* proved suitable for a consortium. *Achromobacter denitrificans* utilized 40.03 mg/L As from the nutrient medium. It also was effective in removing Hg (73.08 mg/L), Pb (71.10 mg/L), and Cd (62.41 mg/L). *Klebsiella oxytoca* utilized 51.14 mg/L Pb, 51.33 mg/L Hg, and 78.02 mg/L Cd. *Rhizobium radiobacter* had the best results for Ni (56.94 mg/L), Hg (61.57 mg/L), and Pb (74.26 mg/L). These microorganisms proved to be biocompatible and did not inhibit each other’s growth. We united the microorganisms into microbial consortiums and tested their ability to synthesize phytohormones. Consortiums D and C synthesized the largest number of phytohormones. They had good prospects for the phytoremediation method, but this fact needs additional investigation. Consortium D had the optimal ratio of *Achromobacter denitrificans*, *Klebsiella oxytoca*, and *Rhizobium radiobacter* as 1:1:2. This consortium removed 56.39 mg/L Cd, 58.03 mg/L Hg, 61.17 mg/L As, 91.13 mg/L Pb, and 98.22 mg/L Ni from the medium with individual heavy metals. The same consortium also utilized 47.33–83.26 mg/L of composite pollutants, which included Pb, As, Hg, Ni, and Cd. Consortium D spotted no significant decrease in biomass accumulation during cultivation in environments with separate and composite heavy-metal-contaminated media. The optimal pH for Consortium D was pH = 7, while the greatest biomass accumulation was observed at 25–30 °C. The resulting consortium is effective against mixed pollution with heavy metals, increasing the phytoremediation ability of plants. It is assumed that Consortium D is also able to increase the establishment of plants in disturbed areas, however, additional experiments are needed to confirm the data. Further research will be aimed at developing a biological product to improve the efficiency of remediation of lands withdrawn from agricultural use.

## Figures and Tables

**Table 1:** Cultural and morphological profile of microorganisms.

Microorganism	Cultural and Morphological Features
1	Mobile, Gram-negative bacilli of 0.5–1.0 × 1.0–3.0 µm that do not form spores; even and round glossy yellow colonies with a flat profile, 1.7–2.1 mm in diameter.
2	Immobile, Gram-positive diplo-bacilli of 1.1–1.6 × 0.5–0.7 μm that do not form spores but have a capsule; even and round glossy yellow colonies with an elevated profile, 0.9–1.2 mm in diameter.
3	Mobile, Gram-negative bacilli of 0.3–0.5 × 0.9–1.2 μm that do not form spores; even and round dull colonies, non-pigmented, slightly convex, 1.0–1.5 mm in diameter.
4	Immobile, Gram-positive bacilli of 0.4–0.7 × 1.5–1.7 µm that do not form spores and have a nucleus; even and round glossy colonies, whitish and flat, 2.1–2.8 mm in diameter.
5	Mobile, Gram-negative bacilli of 0.4–0.6 × 0.3–0.5 µm that form spores; even and round yellow glossy colonies, with an elevated profile, 0.9–1.2 mm in diameter.
6	Immotile, Gram-negative bacilli of 1.3–1.5 × 0.6–0.8 µm that form spores and have a capsule; even and round oily colonies of light beige color, convex, 2.0–3.5 mm in diameter.
7	Mobile, Gram-negative bacilli of 1.5–3.0 × 0.6–1.0 µm that do not form spores; even and round glossy colonies, non-pigmented and convex, 0.9–1.1 mm in diameter.
8	Mobile, Gram-positive diplococci and streptococci of 0.5–0.6 µm that do not form spores; even and rounded dim colonies, transparent and flat, 1.7–2.1 mm in diameter.
9	Mobile, Gram-negative cocci and diplococci of 0.4–0.6 µm that form spores; smooth and round orange oily colonies, with an elevated profile, 0.8–1.0 mm in diameter.
10	Mobile, Gram-negative bacilli of 0.7–0.8 × 2.3–2.8 µm that form spores; even and round glossy colonies, light brown, with an elevated profile, 0.8–1.3 mm in diameter.

**Table 2:** Total absorption of individual metals.

Isolate	Total Metal Absorption, mg/L				
	Pb	As	Hg	Ni	Cd
1	62.15 ± 3.17	37.44 ± 1.65	45.44 ± 2.23	48.10 ± 2.36	57.51 ± 2.65
2	46.02 ± 2.25	25.76 ± 1.03	13.32 ± 0.96	47.41 ± 2.04	19.14 ± 1.14
3	73.10 ± 2.55	40.03 ± 1.60	73.08 ± 3.82	53.32 ± 2.29	62.41 ± 2.99
4	34.76 ± 1.77	33.21 ± 1.43	41.44 ± 1.70	36.08 ± 1.62	38.50 ± 1.85
5	36.02 ± 1.66	28.47 ± 1.31	21.46 ± 1.01	39.74 ± 1.22	16.72 ± 0.87
6	51.14 ± 2.35	44.37 ± 1.86	51.33 ± 2.31	47.32 ± 2.13	78.02 ± 3.67
7	74.26 ± 3.42	59.52 ± 2.86	61.57 ± 3.16	56.96 ± 2.90	41.65 ± 2.04
8	25.11 ± 1.16	24.57 ± 1.03	32.34 ± 1.68	48.19 ± 1.97	29.97 ± 1.44
9	22.04 ± 1.06	10.36 ± 0.41	64.38 ± 2.64	43.50 ± 2.11	11.84 ± 0.50
10	46.36 ± 2.04	43.45 ± 2.16	56.16 ± 2.75	43.32 ± 2.21	53.46 ± 2.19

**Table 3:** Accumulation of biomass in an environment with individual heavy metal.

Isolate	Biomass Accumulation, g/L					
	Pb	As	Hg	Ni	Cd	Control (No Metal)
1	0.88 ± 0.07	0.75 ± 0.07	0.69 ± 0.06	0.81 ± 0.07	0.90 ± 0.08	0.91 ± 0.07
2	0.82 ± 0.07	0.28 ± 0.02	0.21 ± 0.02	0.85 ± 0.07	0.54 ± 0.05	0.88 ± 0.05
3	0.85 ± 0.07	0.72 ± 0.06	0.77 ± 0.07	0.90 ± 0.08	0.87 ± 0.08	0.93 ± 0.05
4	0.63 ± 0.05	0.69 ± 0.06	0.51 ± 0.04	0.55 ± 0.05	0.58 ± 0.05	0.90 ± 0.06
5	0.50 ± 0.04	0.37 ± 0.03	0.41 ± 0.04	0.44 ± 0.04	0.29 ± 0.03	0.94 ± 0.08
6	0.68 ± 0.06	0.93 ± 0.08	0.88 ± 0.08	0.78 ± 0.07	0.86 ± 0.07	0.91 ± 0.06
7	0.81 ± 0.07	0.74 ± 0.06	0.61 ± 0.05	0.89 ± 0.08	0.77 ± 0.07	0.89 ± 0.07
8	0.33 ± 0.03	0.21 ± 0.02	0.48 ± 0.04	0.57 ± 0.05	0.40 ± 0.03	0.87 ± 0.04
9	0.35 ± 0.03	0.19 ± 0.02	0.74 ± 0.06	0.59 ± 0.05	0.22 ± 0.02	0.81 ± 0.05
10	0.82 ± 0.07	0.76 ± 0.07	0.68 ± 0.06	0.91 ± 0.08	0.79 ± 0.07	0.96 ± 0.08

**Table 4:** Physiological and biochemical signs of microorganisms.

Substrate	*Pantoea* sp.	*Achromobacter denitrificans*	*Klebsiella oxytoca*	*Rhizobium radiobacter*	*Pseudomonas fluorescens*
Ala-Phe-Pro-arylamidase	−	−	−	−	−
Adonitol	−	−	+	+	−
L-pyrrolidonyl arylamidase	−	+	+	−	+
L-Arabitol	−	−	−	+	−
D-Cellobiose	+	−	+	+	−
Beta-galactosidase	+	−	+	−	−
H_2_S	−	−	−	−	−
Beta-N-acetyl-glucosaminidase	−	−	−	−	−
Glutamyl arylamidase pNA	−	−	−	−	−
D-glucose	+	−	+	+	+
Gamma-glutamyl-transferase	+	−	+	−	−
Fermentation/glucose	+	−	+	−	−
Beta-glucosidase	−	−	+	+	−
D-maltose	+	−	+	−	−
D-mannitol	+	−	+	+	−
D-mannose	+	−	+	+	+
Beta-xylosidase	+	−	+	−	−
Beta-alanine arylamidase pNA	−	−	−	−	−
L-proline arylamidase	−	−	−	−	+
Lipase	−	−	−	−	−
Palatinose	−	−	+	+	−
Tyrosine arylamidase	−	−	−	+	−
Urease	−	−	−	−	−
D-sorbitol	+	−	+	+	−
Saccharose/sucrose	+	−	+	+	−
D-tagatose	−	−	+	+	−
D-trehalose	+	−	+	+	−
Citrate (sodium)	+	−	+	−	+
Malonate	−	−	+	−	−
5-keto-D-gluconate	−	−	+	−	−
L-Lactate alkalinization	+	+	+	−	−
Alpha-glucosidase	−	−	−	−	−
Succinate alkalinization	−	+	+	−	−
Beta-N-acetyl-galactosaminidase	−	−	−	−	−
Alpha-galactosidase	−	−	+	−	−
Phosphatase	+	−	+	−	−
Glycine arylamidase	−	−	−	−	+
Ornithine decarboxylase	−	−	−	−	−
Lysine decarboxylase	−	−	+	−	−
L-histidine assimilation	−	+	−	−	−
Coumarate	−	−	−	−	+
Beta-glucuronidase	−	−	−	−	−
O/129 resistance (comp. vibrio)	+	−	+	−	−
Glu-Gly-Arg-arylamidase	−	−	−	−	−
L-malate assimilation	+	−	−	−	−
ELLMAN	+	−	+	−	−
L-Lactate assimilation	−	−	−	−	−

**Table 5:** Biocompatibility of the studied microorganisms.

Microorganism	*Pantoea* sp.	*Achromobacter denitrificans*	*Klebsiella oxytoca*	*Rhizobium radiobacter*	*Pseudomonas fluorescens*
*Pantoea* sp.		−	+	−	+
*Achromobacter denitrificans*	−		+	+	−
*Klebsiella oxytoca*	+	+		+	−
*Rhizobium radiobacter*	−	+	+		−
*Pseudomonas fluorescens*	+	−	−	−	

**Table 6:** Total absorption of individual metals by various consortiums.

Consortium	Total Metal Absorption, mg/L				
	Pb	As	Hg	Ni	Cd
A	63.50 ± 3.11	45.08 ± 2.21	35.73 ± 1.75	54.46 ± 2.67	47.13 ± 2.31
B	82.84 ± 4.06	38.46 ± 1.89	63.90 ± 3.04	76.53 ± 3.75	78.60 ± 3.86
C	58.18 ± 2.85	49.42 ± 2.42	54.41 ± 2.67	51.98 ± 2.55	94.50 ± 4.64
D	91.13 ± 4.47	61.17 ± 3.00	58.03 ± 2.85	98.22 ± 4.82	56.39 ± 2.77

**Table 7:** Accumulation of biomass by consortiums in an environment with individual heavy metal.

Consortium	Biomass Accumulation, g/L					
	Pb	As	Hg	Ni	Cd	Control (No Metal)
A	0.87 ± 0.08	0.71 ± 0.06	0.55 ± 0.06	0.94 ± 0.08	0.78 ± 0.07	0.92 ± 0.08
B	0.77 ± 0.07	0.72 ± 0.06	0.74 ± 0.07	0.85 ± 0.07	0.90 ± 0.08	0.91 ± 0.08
C	0.69 ± 0.06	0.90 ± 0.08	0.73 ± 0.06	0.67 ± 0.06	0.92 ± 0.08	0.91 ± 0.06
D	0.91 ± 0.08	0.74 ± 0.06	0.48 ± 0.05	0.71 ± 0.06	0.77 ± 0.07	0.93 ± 0.07

**Table 8:** Biomass accumulation and total absorption of mixture metals by various consortiums.

Consortium	Total Metal Absorption, mg/L					Biomass Accumulation, g/L	
	Pb	As	Hg	Ni	Cd	Composite Pollutant	Control
A	58.48 ± 2.87	41.91 ± 2.06	19.03 ± 0.93	42.81 ± 2.10	38.10 ± 1.87	0.87 ± 0.07	0.90 ± 0.08
B	39.83 ± 3.72	48.01 ± 2.60	23.41 ± 2.33	44.40 ± 4.04	18.95 ± 4.12	0.83 ± 0.08	0.91 ± 0.08
C	46.60 ± 2.29	26.19 ± 1.28	14.65 ± 0.72	21.73 ± 1.07	58.08 ± 2.85	0.85 ± 0.07	0.88 ± 0.08
D	75.07 ± 1.92	53.72 ± 2.39	47.33 ± 1.14	82.95 ± 2.20	83.26 ± 0.90	0.96 ± 0.07	0.90 ± 0.08

**Table 9:** Synthesis of phytohormones by consortia.

Consortium	Indole-3-Acetic Acid, μg/mL of Nutrient Medium	Indole-3-Butyric Acid, μg/mL of Nutrient Medium
A	14.63 ± 0.63	1.93 ± 0.06
B	12.32 ± 0.52	0.63 ± 0.03
C	10.12 ± 0.47	0.97 ± 0.04
D	18.03 ± 0.92	2.02 ± 0.07

**Table 10:** Efficiency of soil phytoremediation when treated with Consortium D.

Seed Soaking	Watering	Residual Content Metal in Soil, %					Average Survival Rate of Seedlings, pcs
		Pb	As	Hg	Ni	Cd	
Consortium D 1.5 × 10^−2^, McFarland standard	Water	77.35 ± 3.01	87.47 ± 2.31	89.66 ± 2.27	76.95 ± 2.21	79.36 ± 2.08	8 ± 1
Consortium D 2.5 × 10^−2^, McFarland standard		68.14 ± 2.96	85.36 ± 1.84	87.21 ± 2.73	69.11 ± 2.85	75.51 ± 2.16	9 ± 2
Water	Consortium D 1.5 × 10^−2^, McFarland standard	79.67 ± 2.71	93.87 ± 2.98	94.47 ± 1.32	79.12 ± 3.03	84.68 ± 2.48	7 ± 2
	Consortium D 2.5 × 10^−2^, McFarland standard	76.33 ± 1.99	89.24 ± 2.04	91,36 ± 2.62	81,87 ± 2.28	83.74 ± 1.74	9 ± 1
Water	Water	84.32 ± 2.21	95.47 ± 1.72	98.36 ± 2.14	86.47 ± 2.55	92.84 ± 1.89	5 ± 2

## Data Availability

The data are available from the authors on request.
